# Gut Microbiota for Esophageal Cancer: Role in Carcinogenesis and Clinical Implications

**DOI:** 10.3389/fonc.2021.717242

**Published:** 2021-10-18

**Authors:** Jianfeng Zhou, Shangwei Sun, Siyuan Luan, Xin Xiao, Yushang Yang, Chengyi Mao, Longqi Chen, Xiaoxi Zeng, Yonggang Zhang, Yong Yuan

**Affiliations:** ^1^ Department of Thoracic Surgery, West China Hospital, Sichuan University, Chengdu, China; ^2^ West China Biomedical Big Data Center, West China Hospital, Sichuan University, Chengdu, China; ^3^ Department of Periodical Press, National Clinical Research Center for Geriatrics, West China Hospital, Sichuan University, Chengdu, China; ^4^ Nursing Key Laboratory of Sichuan Province, West China Hospital, Sichuan University, Chengdu, China

**Keywords:** microbiota, esophageal cancer, carcinogenesis, biomarker, therapeutic complication

## Abstract

Esophageal cancer (EC) is a common malignant tumor of the upper digestive tract. The microbiota in the digestive tract epithelium comprises a large number of microorganisms that adapt to the immune defense and interact with the host to form symbiotic networks, which affect many physiological processes such as metabolism, tissue development, and immune response. Reports indicate that there are microbial compositional changes in patients with EC, which provides an important opportunity to advance clinical applications based on findings on the gut microbiota. For example, microbiota detection can be used as a biomarker for screening and prognosis, and microorganism levels can be adjusted to treat cancer and decrease the adverse effects of treatment. This review aims to provide an outline of the gut microbiota in esophageal neoplasia, including the mechanisms involved in microbiota-related carcinogenesis and the prospect of utilizing the microbiota as EC biomarkers and treatment targets. These findings have important implications for translating the use of gut microbiota in clinical applications.

## Introduction

Esophageal cancer (EC) is one of the most common cancers and is a primary health burden worldwide (1). Globally, among all cancers, EC ranked tenth in incidence (>604,000 new cases) and sixth in mortality (>544,000 deaths) in 2020 (2, 3). Moreover, the incidence of EC in Asian countries is 2.1–16.9/100,000 (4), which is higher than in other regions of the world. EC is composed of esophageal squamous cell carcinoma (ESCC) and esophageal adenocarcinoma (EAC) (1), which are more prevalent in developing and developed countries, respectively (5). As with many diseases, several genetic and environmental factors play key roles in the formation and progression of EC (6). The exact cause of EC is unknown, but smoking and heavy drinking have been proven to be important causes (7, 8). Recently, Kamangar et al. reported the impact of hot tea on esophageal carcinogenesis (9). These studies reveal that the environment plays a significant role in EC.

Gut microbiota principally refers to the microorganisms (mainly comprising bacteria) that dwell in the digestive tracts of humans (10), principally including the esophageal, oral, and intestinal microbiota (11–13). In the past few years, the role of gut microbiota in cancer environmental factors has become an increasing concern. Interestingly, a recent study reported that infectious etiology accounts for approximately 15% of all cancer cases, such as gastric cancers caused by *Helicobacter pylori* and hepatocellular carcinomas caused by hepatitis viruses (14). In addition to specific infectious microorganisms, several researchers have started to focus on the symbiotic microbial community, which may lead to tumorigenesis. In the case of the esophagus, these microorganisms are important to esophageal physiology such as metabolism (15) and immune maturation (16), and changes in their relative abundance can disrupt their balanced interaction with the esophagus, leading to esophageal diseases, such as EC (17). In this review, we summarize the relationships between gut microbiota and EC and evaluate their potential for clinical applications.

## Microbiota in Esophageal Cancer and Detection Methods

Human studies currently use comparative metagenomic approaches to explore the role of microbiota in EC. However, in the 1980s, traditional bacterial culture methods were still dominant in microbiology research (18), and early studies reported that there are a substantial number of microorganisms in many microenvironments that cannot be cultured (19).

Later, metagenomic studies have used non-culture methods to characterize the diversity of microbial flora, and these methods have higher sensitivity and specificity (20). 16S ribosomal RNA (16S rRNA) sequences have emerged because they can detect the previously unknown species, indicating that cultivation-based methods find less than 1% of the microbiota in samples (21). At present, the most commonly used technique in metagenome research is also the sequencing of the 16S rRNA gene, which is located in the highly conserved region of all bacteria (22). After amplification with universal primers, the read sequence is compared with the database of known 16S rRNA gene sequences, which can be used to classify and identify the bacteria in the sample (23), thus, 16S rRNA sequencing technology notably reduces the cost of identifying the composition of esophageal microorganisms, making large-scale research possible (24).

Recently, researchers are no longer satisfied with measuring just a tiny piece of a microbial genome. Metagenomic sequencing, which sequenced the whole genome of microorganisms, emerged. In 2002, Breitbart et al. first used environmental metagenomic sequencing to demonstrate that seawater contains over 5,000 different viruses (25). Metagenomic sequencing can provide complete information on the whole genome by randomly fragmenting all the DNA of the microbiota in samples into small segments; then, they are assembled together based on the overlapping ends into longer fragments (26). Moreover, the complete genome information can be used to metabolic capacities of the microbiota in the samples, which makes it is more valuable than the 16s rRNA sequencing.

The common feature of these two methods is that they use sequencing technology to detect the DNA sequence of microorganisms in the samples, which they compare with the database of known sequences to determine the existence and abundance of specific microorganisms (22). Recently, a large number of human metagenomic sequencing or 16S rRNA sequencing studies have been conducted to describe the EC microflora of esophageal tissues and stool and saliva samples (20). A growing number of studies have revealed that the gut microbiota related to EC differs from the microbiota of healthy subjects (27–30).

### Esophageal Microbiota in Esophageal Adenocarcinoma

It is well known that gastroesophageal flux disease (GERD) complicating Barrett’s esophagus (BE) eventually leads to EAC (5). Approximately 10% of GERD patients develop BE, and 1% of them will most likely develop EAC (31). In a meta-analysis, the occurrence of GERD symptoms at least weekly increased the risk of EAC approximately five times (32).

In this context, several studies have investigated the esophageal microbial community in patients with BE. For example, Macfarlane et al. found distinct differences in esophageal microbiota samples between BE patients and normal individuals (33). The study showed that more types of bacteria were isolated from patients with BE than from patients without BE; for example, *Campylobacter* was abundant in patients with BE, whereas it was not identified in normal individuals (33). This indicates that patients with BE had higher microbial diversity. Furthermore, using 16S rDNA technology, Yang et al. investigated the esophageal microflora of patients with a normal esophagus, reflux esophagitis (RE), and BE. According to the sequencing results, they divided the esophageal microflora into type I and type II microflora. Comprised mainly of Gram-positive bacteria such as *Firmicutes*, type I microflora are related to the normal esophagus. Type II microflora are mainly associated with RE and BE and comprise a large number of Gram-negative anaerobic/aerobic microorganisms such as *Bacteroides*, *Proteus*, *Clostridium*, and *Spirillum* (27). The transition from Gram-positive aerobic microflora to Gram-negative anaerobes may be related to changes in the microenvironment and a disease state (27). Moreover, Liu et al. also compared the 16S rDNA results of esophageal bacteria in patients with RE and BE and normal individuals, and found that *Clostridium welchii*, *Prevotella*, *Neisseria*, and *Clostridium* were more common in RE and BE patients than in normal people (34). In addition, Yang et al. showed that the number of bacteria in the normal, esophagitis, and BE groups was similar and the change was the relative abundance of bacteria (35). These studies suggest that the composition of esophageal microflora in normal esophagus, RE, and BE is different, indicating that esophageal diseases may be associated with the microflora structure.

In contrast to GERD and BE, the microbial diversity of the esophagus with EAC decreased compared with normal controls because of the abundance of *Lactobacillus* and some other bacteria (36). The development of EAC and the lactic acid produced by *Lactobacillus* may acidify the internal environment of the esophagus. In addition, hydrogen peroxide, a toxic product of these bacteria, may directly inhibit the growth of other bacteria, making the lower part of the EAC esophagus to be dominated by *Lactobacillus* (36). Therefore, abundant *Lactobacillus* may be applicable to early EAC detection. In a study published in 2019, samples were collected from 16 normal individuals, 14 patients with non-dysplastic BE, 10 patients with dysplastic BE, and 4 patients with EAC, and the microbiota changes were apparent in cases of low-grade dysplasia and EAC, where the proportion of *Firmicutes* (mainly *Streptococcus*) in low-grade dysplasia was higher than that in adenocarcinoma (37).

Apart from the differences in microbial diversity, studies have also focused on the functions of different microorganisms. For instance, Lopetuso et al. found distinct functions for both BE and EAC groups. On the one hand, the microorganisms have a high tendency for replication and repair in BE, while on the other hand, there is an upregulated potential for energy, replication, and signaling metabolism and a downregulation trend of the fatty acid biosynthesis and nitrogen and D-alanine pathways in EAC (38). Overall, the EAC microbiota and associated precancerous lesions exhibit an apparent compositional and metabolic function shift compared with the microbiota of healthy individuals, reflecting a different ecological microenvironment of the esophagus in patients with EAC. Hence, it is plausible to suggest that the change in microflora may be related to the development of EAC.

### Esophageal Microbiota in Esophageal Squamous Cell Carcinoma

It is now well established that esophageal dysplasia is a precursor for the majority of ESCC (39). As most cases of ESCC begin with malignant transformation of dysplasia, a growing number of studies have attempted to detect the microbiota of esophageal dysplasia and ESCC. A study published in 2014 gathered samples from 192 subjects without esophageal squamous dysplasia and 142 patients with esophageal squamous dysplasia and showed a significant difference in microbiota between these two groups, where lower microbial richness was associated with the presence of esophageal squamous dysplasia (40). Moreover, Li et al. found a significant increase in *H. pylori* infection in esophageal tumor tissues (including ESCC and samples adjacent to the ESCC) compared with that in non-tumor tissues. The 16S rRNA-positive rate of *H. pylori* in ESCC, samples adjacent to the ESCC, and normal samples were 62.5, 74.1, and 26.7%, respectively (41). This study suggested that the high level of *H. pylori* in the esophagus may be related to the development of ESCC. Gao et al. found a special infection of *Porphyromonas gingivalis* in the esophageal tissues of patients with ESCC, but the infection was unnoted in healthy tissues of the control group, supporting the vital pathogenic role of *P. gingivalis* in ESCC (28). In addition, Shao et al. found that the microbial environment of ESCC tissues was primarily composed of *Firmicutes*, *Bacteroidetes*, and *Proteobacteria*. ESCC tumor tissues contained more *Fusobacterium* and less *Streptococcus* than non-tumor tissues (42). These differences offer proof of the key role of the microbiota in carcinogenesis and reveal some underlying communities of microorganisms that are likely carcinogenic.

In addition, microbial imbalances can induce further systemic metabolic changes (43, 44). For instance, Li et al. found that compared with the healthy control group, the pathways related to the metabolism of cysteine, methionine, fructose, galactose, and starch, as well as the pathways related to DNA repair and recombination, protein translation, chromosomal dynamics, and peptidase activity were upregulated in the ESCC group (45). Moreover, a study published in 2021 in which samples were collected from 18 patients with ESCC and 11 normal subjects revealed that the ESCC microbiota had altered nitrate and nitrite reductase activities compared with the normal control group (46). Animal studies in ESCC mice also showed changes in esophageal metabolism; for instance, Cheung found a decrease in *Pasteurellales* and upregulated metabolic pathways relevant to carbohydrate and lipid metabolism in a xenograft mouse model of ESCC cells (47). Consequently, there is a reason to believe that changes in these metabolic pathways caused by the ESCC microbiota might be closely related to the appearance and development of esophageal tumors. Therefore, we believe that the change from normal microbiota to ESCC microflora is closely related to the occurrence and development of ESCC.

### Oral and Intestinal Microbiota in Esophageal Cancer

In addition to the microbiota in the esophagus itself, the oral and intestinal microbiota are vital in the development of the tumor. For instance, Snider et al. found that at the phylum level, there were distinctly enhanced relative loads of *Firmicutes* and decreased relative loads of *Proteobacteria* in BE patients (29). This reveals that the oral microbiome in BE patients was significantly changed, which might be related to the progress of BE. Furthermore, Peters et al. found that the composition of oral microflora can reflect the potential risk of EC; *Neisseria* and *Streptococcus pneumoniae* were positively related to the presence of EAC, and a load of *P. gingivalis* was consistent with a high risk of ESCC (48). In addition, a study published in 2015 collected saliva samples from 87 incident and histopathologically diagnosed ESCC cases, 63 dysplasia cases, and 85 non-disease cases and reported the existence of obvious oral microbiota shifts from normal oral microbiota to ESCC microflora (30). Therefore, proposing a prediction model for the change in the corresponding microflora may provide clues regarding the early detection and diagnosis of EC. In addition, Qian et al. collected saliva from 20 patients with ESCC and 21 healthy controls and found that the healthy control group was positively related to *Fusobacterium* and *Porphyromonas*, while the susceptibility of ESCC was possibly correlated to *Actinomyces and Atopobium* (49). Futhermore, a study published in 2019 by Kageyama reported that there was more *P. gingivalis* in the saliva of EC patients relative to that in the control subjects (50); *P. gingivalis* might play the same role in EC as hepatitis viruses in hepatocellular carcinomas. In addition, Zhao et al. found that in 39 EC patients, the most significantly increased taxa were *Firmicutes*, *Negativicutes*, and *Selenomonadales*, compared with 51 healthy subjects (11). Above all, the oral microbiota in EC patients exhibit a shift relative to the microbiota of healthy individuals, and these changes lay the foundation for the use of oral microbiota as biomarkers for the early diagnosis of EC.

In addition, several studies have shown the intestinal microbial structure in patients with EC. For instance, Tanaka et al. reported that obligate anaerobes, such as the *Clostridium coccoides* and *Bacteroides fragilis* groups, were predominant in patients with esophageal tumors (51). Moreover, a study published in 2015 collected stool samples from 31 subjects with advanced EC revealed that *the Clostridium leptum* subgroup and *Clostridium coccoides* group dominated the community of gut microbes in EC participants (12). These studies suggested that the gut microbiota related to EC was characteristic, enabling them to be latent biomarkers for early EC diagnosis.

## Mechanisms in Carcinogenesis

The number of genes in the gut microflora is approximately 100 times that in the human genome (52). Although the microbiota is most dense in the lower intestine, its influence on host immunity extends beyond the lower intestine (53). These genes provide complex tools that enable microbiota to use digestive tract substances to adapt to host defense immunity, inflammation, tissue development, and substance metabolism, and interact with the host to form symbiotic networks (54, 55). Esophageal carcinogenesis has a complicated course that is affected by genetic and environmental factors (56). Some of these carcinogenesis-related mechanisms, including inflammation and immune regulation, microbial components, and the production of genotoxins, are closely linked to the gut microbiota.

### Inflammation and Immune Regulation

Several studies have reported that the host immune system play a vital role in the carcinogenesis of gastrointestinal cancer (57). Gut microbiota could not only affect the local immunity of the digestive tract mucosa but also initiate the systemic immune response through whole-body immune cells (58). Furthermore, microflora and immune disorders may lead to inflammation, and consistent chronic release of inflammatory mediators is usually a initial contributing factor of cancer (59). The important influence of the microflora on the host immune system derives from the study of aseptic animals, which lack intestinal microflora (54, 60). Lee et al. found defects in the development of the immune system of these mice, which was characterized by a decrease in the number of CD4+ T cells and a corresponding decrease in the expression of Toll-like receptors (TLRs) and MHC II in their intestinal epithelial cells (54, 60). Therefore, normal microorganisms may play an important role in the development of immunity. On the other hand, when the balance between the microflora and the body’s immune system is disrupted, the immune system’s response to prevent bacterial invasion may trigger tumor growth (17). For example, Boursi et al. found that disorders of the microflora and the immune system caused by penicillin were associated with EC or precancerous lesions (61).

**Figure 1:**
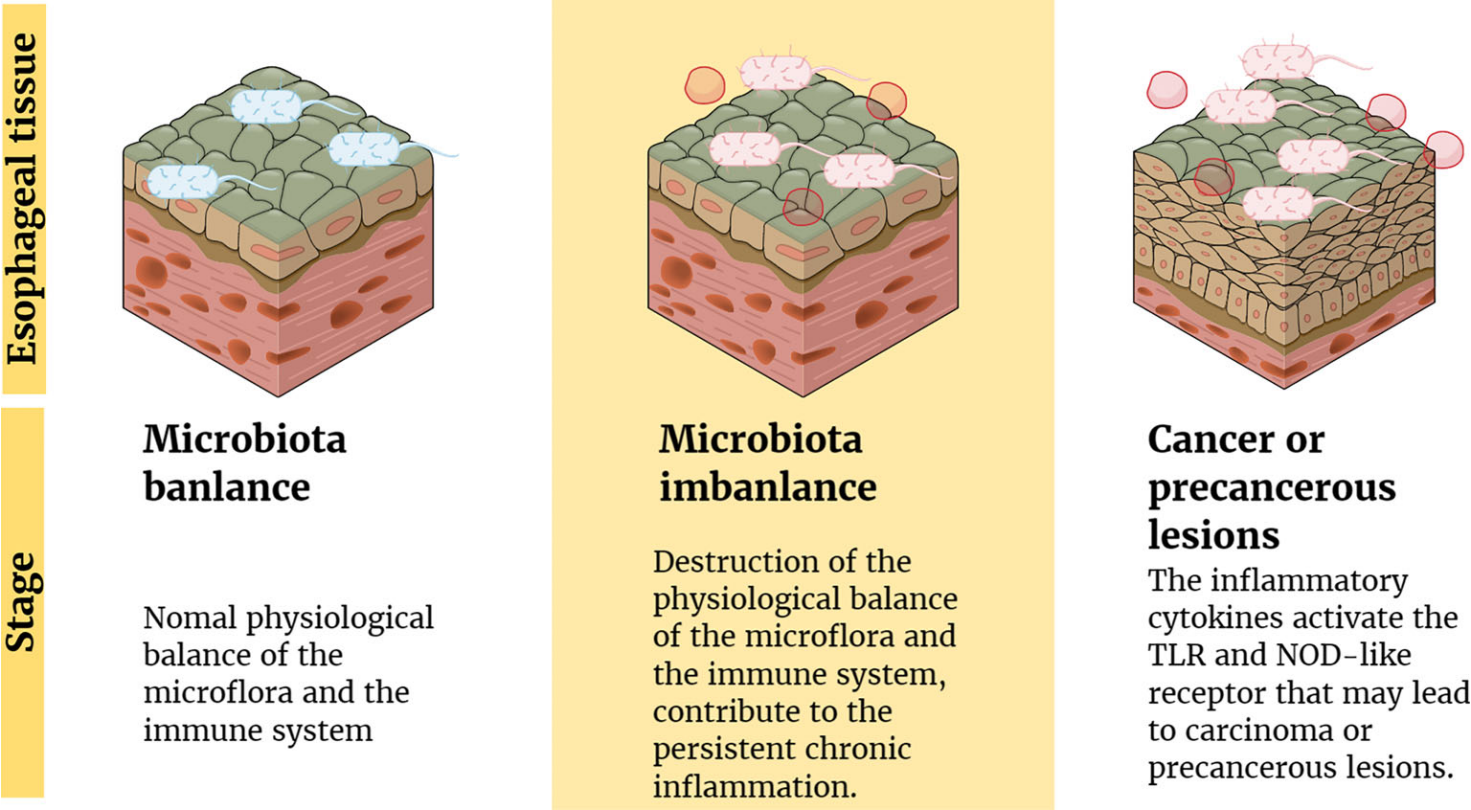
Inflammation and immune regulation mechanism involved in the pathogenesis of EC. Factors (such as antibiotics) lead to the destruction of the physiological balance of the microflora and the immune system, which contribute to the persistent chronic inflammation. Moreover, the inflammatory cytokines activate the TLR and NOD-like receptor that may lead to carcinoma or precancerous lesions.

Several studies have suggested possible mechanisms by which microflora interact with the body’s immune system and signaling pathways to cause cancer. The imbalance between human microflora and the immune system may change the composition of normal microflora in the esophagus and form a microbial-related molecular model (59), including TLR and nucleotide-binding oligomeric domain (NOD)-like receptors (62, 63). Pursuant activation of related receptors might result in the production and release of cytokines and chemokines involved in chronic inflammation to promote the occurrence and progress of cancer (64). For example, BE epithelial biopsies have high levels of proinflammatory cytokines, especially IL-1β (62). Moreover, some inflammatory factors released in the inflammatory condition (such as IL-6 and IL-23) increase the reaction between microorganisms and the host by promoting the inflammatory response, which causes cancer in the end (59). These findings confirm the potential role of inflammation and immune regulation in the development of EC (**Figure 1**).

### Microbial Components

**Figure 2:**
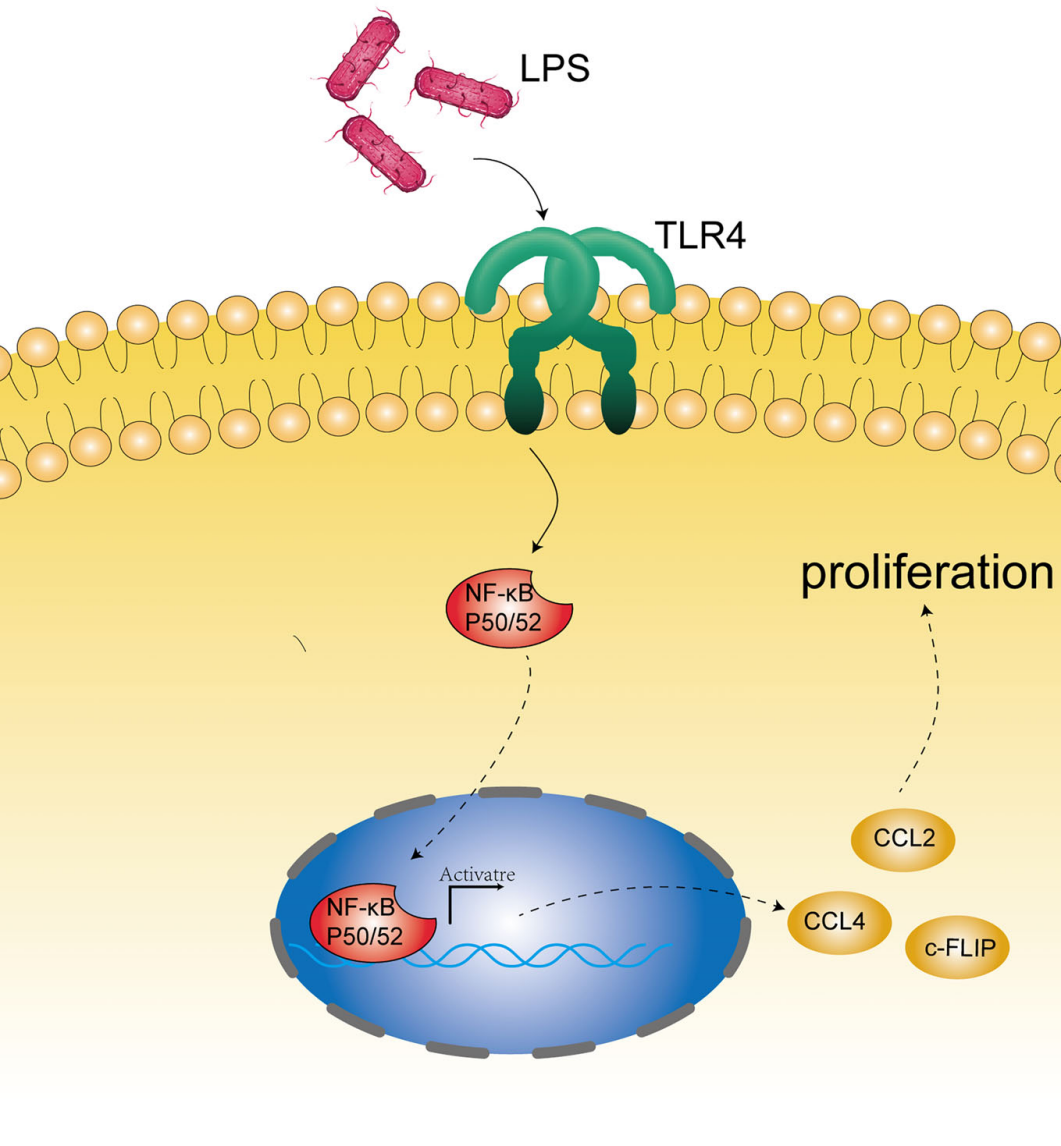
Microbial component mechanism involved in the pathogenesis of EC. Bacterial constituents, such as bacterial cell wall components (mainly LPS) and DNA, may stimulate the receptors on the epithelium (such as TLR4) and activate the NF-kappa B pathway associated with carcinogenesis.

In addition to the effects of microbial disorders on the human immune system, bacterial material, such as bacterial cell wall components and DNA, can be used as ligands for some receptors on the esophageal epithelium. Esophageal type II microflora, dominated by Gram-negative bacteria, can produce abundant lipopolysaccharide (LPS) (27, 65). LPS can delay gastric emptying through cyclooxygenase 1/2 or directly affect the function of the lower esophageal sphincter, and then increase intragastric pressure to promote the occurrence of GERD, leading to the development of EAC (65, 66). Interestingly, as a natural ligand of LPS, the expression of TLR4 increases in the esophageal epithelium of patients with BE and EAC (65). The activation of TLR4 receptor triggers the NF-kappa B pathway associated with inflammation-related carcinogenesis (67) and mediates the formation of early BE (68). Therefore, inflammation and malignant transformation of the esophagus may be accomplished through the activation of the LPS-TLR4-NF-κB pathway (65, 69). In addition, Nadatani et al. found that after BE cells were treated with LPS, the expression of NOD-like receptor protein 3 (NLRP3), the activity of caspase-1, and the secretion of IL-1β and IL-18 increased, which they thought were due to the activation of reactive oxygen species (ROS) by LPS, and these promoted the development of cancer (70) (**Figure 2**).

Moreover, Baba et al. found that in ESCC tissues with *Fusobacterium nucleatum*, the number of specific chemokine (CCL20) genes increased, which indicated that *F. nucleatum* might promote the invasive behavior of esophageal tumors by motivating chemokines (such as CCL20) (71). It is well known that the vital roles of chemokines and their receptors in tumor development and progression in several types of cancers (72). As to their results, the most upregulated chemokine in *F. nucleatum* positive ESCC is CCL20. Remarkably, an increasing number of studies have recently drawn attention to the association of CCL20 and its receptor CCR6 in the oncogenesis of various types of cancers (73). For instance, Wang et al. have reported that CCL20 stimulation promoted cancer cell proliferation and migration *in vitro* (74).

### Production of Genotoxins

Other than the components of the microbiota itself, some bacteria may directly produce genetic toxins or cancer-promoting metabolites that may cause genome damage and lead to EC. For example, cellular lethal swelling toxins secreted by Gram-negative bacteria may cause host DNA damage (75, 76), and further repair of DNA damage might lead to the development of EC. For instance, Gabriel et al. reported that the exposure of cultured mammalian epithelial cells to *E. coli* induced a DNA damage response followed by cell division with signs of incomplete DNA repair, leading to anaphase bridges and chromosome aberrations. Exposed cells exhibited a significant increase in gene mutation frequency and anchorage-independent colony formation, demonstrating the infection mutagenic and transforming potential (76).

**Figure 3:**
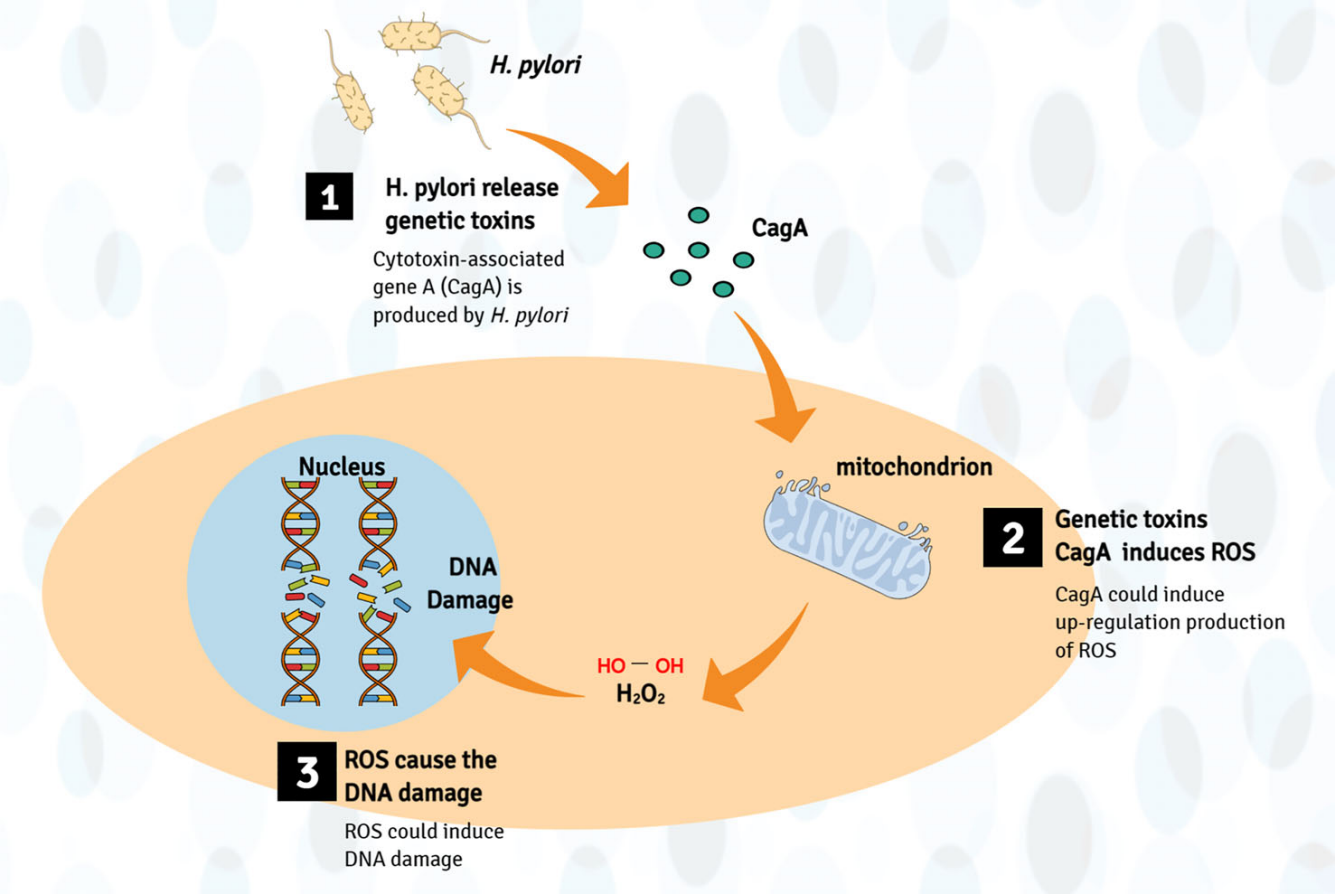
Production of genotoxins mechanism involved in the pathogenesis of EC. Bacteria (such as *H. pylori*) may directly produce genetic toxins (mainly CagA) or cancer-promoting metabolites, which may cause genome damage by upregulating the production of ROS, leading to esophageal cancer (host DNA damage).

In addition, cytotoxin-associated gene A (CagA) and vacuolating cytotoxin A produced by *H. pylori* can stimulate inflammation and lead to the development of cancer (77). CagA is a cancer-related protein, which could induce DNA damage through host-mediated upregulation* *production of ROS (78, 79). Moreover, vacuolating cytotoxin A can change membrane permeability and lead to apoptosis rates increase (80). For instance, Li et al. found that CagA1-positive *H. pylori* can cause DNA breaks in esophageal epithelial cells, which can lead to atypical hyperplasia of esophageal squamous epithelial tissues and contribute to the carcinogenesis of ESCC. Further mechanism research found that *H. pylori* infection induces ROS in the cytoplasm, which promotes the DNA damage response (41) (**Figure 3**).

Generally, inflammation, the immune response, microbial components, and toxic products are the main mechanisms by which the gut microbiota promotes malignant transformation of the esophagus.

## Using the Microbiota as Biomarkers

One growing translational application of the digestive tract microbiota is to use them as biomarkers for screening and prognosis prediction. Accumulating evidence suggests that precise and convenient screening tests could notably decrease the global burden of EC (81). Moreover, several studies have reported the relationship between specific bacterial markers and clinical outcomes. In this section, we discuss microbiota-related biomarkers (mainly oral and esophageal microbiota) for EC screening and prognosis prediction (**Table 1**).

**Table 1:** Gut microbiota biomarkers for EC screening and prognostication.

Study	Population(s)	Study sample size	Study period	Study platform	Main findings	Marker type
Blackett et al. (82)	British	37 GERD, 45 BE, 30 EAC, and 39 healthy controls	2013	qPCR	The abundance of *Campylobacter* in esophageal adenocarcinoma was significantly lower than that in GERD and Barrett’s esophagus.	Diagnosis
Chen et al. (30)	Chinese	87 ESCC and 85 healthy controls	2015	16S rRNA	*Prevos, Pseudomonas, and Streptococcus* had a higher abundance in saliva than the control group in ESCC.	Diagnosis
Elliott et al. (36)	British	19 EAC and 20 healthy controls	2017	16S rRNA	*Lactobacillus fermentum* was enriched in esophageal adenocarcinoma.	Diagnosis
Peter et al. (48)	American	81 EAC and 160 healthy controls 25 ESCC and 50 healthy controls	2017	16S rRNA	*Neisseria and Streptococcus pneumoniae/Porphyromonas gingivalis* were positively related to the presence of EAC/ESCC, respectively	Diagnosis
Snider et al. (29)	American	32 BE and 17 healthy controls	2018	16S rRNA	A model including relative abundance of *Lautropia*, *Streptococcus*, and *Bacteroidales* in order to distinguish BE from controls with an area under the ROC curve of 0.94	Diagnosis
Zhou et al.	Australian	6 EAC and 16 healthy controls	2020	16S rRNA	A high abundance of *Staphylococcus*, *Lactobacillus*, *Bifidobacterium*, and *Streptococcus*, which contribute towards dysregulated lactic acid-producing, consist of a distinct EAC microbiota.	Diagnosis
Yang et al. (46)	Chinese	18 ESCC and 11 healthy controls	2021	16S rRNA	Employing decreased abundance of *Bacteroidetes, Fusobacteria*, and *Spirochaetes* into a microbial dysbiosis index showed that dysbiosis microbiota had an excellent capacity to discriminate between ESCC and normal subjects.	Diagnosis
Deng et al. (83)	Chinese	23 EC and 23 healthy controls	2021	16S rRNA	ROC analysis revealed that *Lachnospira*, *Bacteroides*, *Streptococcus*, and *Bifidobacterium* achieved an area under the curve that was more than 0.85, showing high accuracy in EC diagnosis	Diagnosis
Gao et al. (28)	Chinses	100 ESCC	2016	qPCR	*Porphyromonas gingivalis* was also positively correlated with the severity and poor clinical prognosis of ESCC.	Prognosis
Liu et al. (84)	Chinses	45 ESCC	2018	16S rRNA	The abundances of genera *Prevotella* and *Streptococcus* were inversely correlated with survival rates in ESCC patients	Prognosis
Yamamura et al. (85)	Japanese	551 ESCC	2019	qPCR	High level of *Fusobacterium nucleatum* in the tumor was associated with larger tumor size, higher T stage, higher TNM stage, and worse prognoses.	Prognosis
Li et al. (45)	Chinese	17 ESCC, 15 patients at 9–12 months after ESCC radical esophagectomy and 16 healthy controls	2020	16S rRNA	*Bacteroidetes* and *Pseudomonas* were the key taxa contributing to the changes in the microbiome of the ESCC and post-ESCC groups which were similar to the healthy control group. The monitoring of the *Bacteroidetes* and *Pseudomonas* may be an essential method to predict the recurrence of the tumor.	Prognosis

### Biomarkers for Screening

It is necessary to identify precise biomarkers for screening early EC that can be cured with favorable clinical outcomes. Combining culture and non-culture methods, Blackett et al. compared the microbiota of a control group with those of patients with GERD, BE, and EAC. They found that the *Campylobacter* load in EAC was significantly lower than that in GERD and BE (82, 86). In addition, the expression of carcinogenesis-related cytokines, such as IL-18, was higher in *Campylobacter-*colonized tissues (82, 86). Considering the potential effect of *Campylobacter* on human tumorigenesis (33), its role in the progression of EAC may be similar to that of *H. pylori* in gastric cancer. Consequently, it is reasonable to believe that *Campylobacter* may be a biomarker of EC. In addition, Elliott et al. found that *Lactobacillus fermentum* was enriched in EAC and the abundance of *Lactobacillus* was closely related to the development of EAC (36). Moreover, Zhou et al. reported a unique EAC microbiota containing a high abundance of *Staphylococcus*, *Lactobacillus*, *Bifidobacterium*, and *Streptococcus*. Their results suggested these microbiotas can be diagnosis biomarkers because of the close relationship between them and the appearance of EAC (87).

In addition to the microflora of the esophagus itself, oral microbiota serves as biomarkers for the early diagnosis of EC. The esophageal microbiota are largely affected by the oral microflora, which might be due to their close proximity anatomically, and the composition of the oral microbiota can provide some evidence of the progress of EC (53). Peters et al. found that the composition of oral microflora can reflect the potential risk of EAC, and according to their conclusion, *Neisseria* and *S. pneumoniae*/*P. gingivalis* were positively related to the presence of EAC/ESCC, respectively (48). Moreover, Chen et al. found that *Prevos*, *Pseudomonas*, and *Streptococcus* were more abundant in the saliva of patients with ESCC than in that of a normal group (30). Furthermore, a study published in 2018 constructed a model including the relative abundance of *Lautropia*, *Streptococcus*, and *Bacteroidales* in order to distinguish BE from controls, which has an area under the SUV curve of 0.94 (29).

### Biomarkers for Cancer Prognostication

Apart from the potential capability of microbiota for EC diagnosis, combinations of microbial biomarkers and clinical outcomes of EC have allowed the use of microbiota as prognostic markers.

Among all the microorganic candidates, *F. nucleatum* seems a crucial prognostic marker. Yamamura et al. found that a high level of *F. nucleatum* in the tumor was associated with a larger tumor size, higher T stage, and higher TNM stage. In addition to being associated with EC staging, they also noted that the total load of *F. nucleatum* in tumor tissues of patients with recurrent EC was significantly higher than that of patients without EC recurrence (85). Moreover, the prognosis was significantly worse in patients with a high load of *F. nucleatum* (85). Furthermore, Gao et al. found that the load of *P. gingivalis* was positively associated with the progression and poor clinical prognosis of ESCC (28). Therefore, *F. nucleatum* and *P. gingivalis* are possible prognostic markers of ESCC.

It has also been reported that multiple microbiota could be combined to form a novel indicator of EC outcomes, and Liu et al. found that the T3-4 group of EC had an increased abundance of *Streptococcus* compared to T1-2. Likewise, the N staging showed no exception; they found that patients with lymph node metastasis had a higher abundance of *Prevotella* and *Treponema* compared to control subjects (84). They also investigated the impact of the microbiota on the long-term prognosis of patients undergoing EC surgery and reported that the abundance of the genera *Prevotella* and *Streptococcus* was inversely correlated with survival rates in ESCC patients, suggesting that a high abundance of these genera predicts poor prognosis. Therefore, they proposed a new index to estimate prognosis based on a combination of the *Streptococcus* and *Prevotella* load (84).

Collectively, the above studies show that the gut microbiota play a key role in the screening of early EC patients and are able to represent the clinical outcome of EC patients. These studies reveal the potential of microbiota to serve as specific biomarkers for screening and prognosis. More clinical trials are needed to accelerate the clinical application of basic experiments related to gut microbiota.

## Modulating Microbiota for Esophageal Cancer Prevention

Preventive measures are an attractive strategy for reducing the burden of EC. Extensive epidemiological studies have identified several risk factors for EC, including dietary patterns, obesity, and other lifestyle factors, which may be readily modifiable (88). In addition, antibiotics (89) and probiotics (90) have been studied in the context of EC prophylaxis. Here, we review the potential of these factors to reduce EC by regulating gut microbiota.

### Administration of Probiotics

Probiotics are live microbes that benefit health by improving the gut microbiota after being administered (91). The first discovery of probiotics was made in 1905 (92); recently, the anticancer activities of these microorganisms and their potential immune mechanisms have aroused interest (93). For EC, several probiotics including *Bifidobacterium* and *Lactobacillus* spp. have shown anticancer activities in clinical studies, for example, Zhang et al. reported that fermented dairy foods, which are known to have probiotic content (such as *Bifidobacterium*), intake decreased EC risk significantly (90). However, the specific mechanisms supporting probiotics to prevent EC are still lacking. More *in vivo* and *in vitro* studies are needed to help determine the role of probiotics in preventing EC.

### Exposure to Antibiotics

It is well known that antibiotics play a crucial role in regulating the gut microbiota (94, 95). Exposure to antibiotics during treatment for infectious diseases may have dramatically changed the gut microbial compositions, which are related to differing EC risks.

Several studies have made progress in the effects of administrating antibiotics or not on EC risk in general. Conflicting results were reported with observational data showing an increase (61) or no change (89) in risk of EC after antibiotic consumption. In detail, Akinari et al. investigated whether alteration of microbiota using penicillin G and streptomycin affects EAC development. As to their study, incidence rates of BE and EAC were no statistical difference between antibiotic and control groups, although the antibiotics group has a trend to reduced incidence of EAC and the esophageal microbiome was different between the two groups (89). Conversely, a study with 125,441 cases and 490,510 matched controls was analyzed by Ben et al. For gastrointestinal malignancies, the use of penicillin was associated with an elevated risk of esophageal, gastric, and pancreatic cancers (61). Therefore, studies with a higher quality of evidence need to be conducted to elucidate the preventive effect of antibiotic regulation of gut microbiota on EC

### Dietary Interventions

Diet is a significant determinant of gut microbes (96). People who ate different diets had significantly different gut microbiome compositions associated with different EC risks. Given that dietary interventions (particularly fiber and fat intake) can profoundly reshape our microbiota (97), interest in dietary interventions to influence the occurrence and progression of gut microbiota to prevent EC has been piqued.

That dietary fat intake can dramatically affect the composition of the gut microbiota has been proved in mouse models (98). Natasha et al. reported that BE model mice fed an HFD developed esophageal dysplasia and tumors more rapidly than mice fed the control diet, which was associated with a shift in the gut microbiota. They observed similar differences in the microbiomes from patients with BE who progressed to EAC and those who did not develop into cancer (99). Besides, Jeffrey et al. demonstrate that chronic HFD alone induced esophageal inflammation and metaplasia *via* increased microbiota diversity (100).

In addition, studies on individual dietary composition have indicated that dietary fiber, which can be acquired from natural food products or supplemented as a prebiotic preparation, is an essential factor affecting gut microbial diversity (77). Prebiotics are compounds in foods that induce the growth or activity of beneficial microorganisms such as bacteria by altering the microbial composition of the gut microbiome (101). Dietary fiber intervention could increase *Bifidobacterium* and *Lactobacillus* spp.’s abundance and boost fecal butyrate concentration in humans (102) through microbial fermentation. Importantly, Nobel et al. collected esophageal samples from 47 ambulatory patients completed a validated food frequency questionnaire quantifying dietary fiber and fat intake to determine the association of the composition of the esophageal microbiome and fiber intake. Their results showed that increasing fiber intake was significantly associated with the increasing relative abundance of *Firmicutes*. Therefore, dietary fiber intake was an essential modifier of the esophageal microbiome, which has the potential to prevent esophageal diseases (103).

### Weight Reduction

Obesity is an assured risk factor for EC (especially EAC), and there seemingly is a linear association between increased body mass index and EAC (104). In addition, multivariable analysis showed that systemic or central obesity was an independent risk factor for EAC (105). Obesity may cause EC through such mechanisms as insulin and insulin-like growth factor signaling, chronic inflammation, and adipokines (106). In addition, the gut microbiota has emerged as a novel mechanism that regulates systemic exposure to bacterial LPS, secondary to changes in intestinal permeability, leading to metabolic disorders, insulin resistance, and thereby promoting EC formation (106, 107).

Previous studies have reported that obesity is associated with reduced microbial diversity (108) and changes in gut microbiome composition (109). Thus, weight control in obese individuals can profoundly alter their gut microbiota (110). Obese individuals who continued to lose weight after bariatric surgery had a lower risk of developing obesity-related cancers than matched obese controls (111), although this study lacked the ability to assess the risk of specific cancer types. Therefore, studies with large sample sizes are urgently needed to specifically clarify whether weight reduction could affect the incidence of EC by altering the gut microbiota.

## Harnessing Microbiota for Esophageal Cancer Therapy-Related Applications

The composition of gut microbiota can be changed by antibiotics (112), prebiotics (113), probiotics (114), or microflora transplantation (115). The treatment of EC by regulating the microflora may become a novel treatment method. Narrow-band antibiotics can selectively remove or inhibit harmful components of the human gut microbiota. Prebiotics can promote the proliferation of beneficial microorganisms. Probiotics can introduce beneficial microbial components that are not present in human hosts. Fecal microflora transplantation may target microflora to treat cancer. However, there are still no reported cases of intestinal microflora use to treat EC directly. Therefore, most applications of gut microbiota are to strengthen the efficacy of neoadjuvant or adjuvant treatment and to prevent complications caused by therapeutic methods.

### Harnessing Microbiota to Enhance the Efficacy of Treatment

In addition to its roles in the direct prevention of EC, there is growing evidence that the gut microbiota could mediate the efficacy of chemotherapy and immunotherapy. This enables their use as biomarkers to predict treatment response and, at the same time, to be modulated to enhance cancer treatment efficacy.

A growing body of evidence suggests that the gut microbiota can adjust the anticancer chemotherapeutic effects by mechanisms of microbial immunomodulation, metabolism, and translocation (116). For instance, it has been reported that the microbiota can improve the efficacy of oxaliplatin because the gut microbiota stimulate immune cells to produce ROS, which enhance the DNA damage caused by oxaliplatin, leading to cell necrosis (55). However, a study that included 30 patients assigned to administrate synbiotics (combination of probiotic and prebiotic) in the course of chemotherapy and 31 control subjects was reported by Masaaki et al. The clinical response rate was 60 and 52% after chemotherapy to synbiotics and control group, respectively. No significant difference but a tendency that synbiotics could promote the effect of chemotherapy was observed in the response rates of the two groups. Since the sample size of the present study was small, larger-scale studies need to demonstrate whether the synbiotics could improve chemotherapy in the future (12). Furthermore, Cheung et al. investigated the association of gut microbiota and EC treatment efficacy by FMT. As to their results, healthy mouse stools did not significantly affect anti-EC medicinal herb Andrographis paniculata (AP) efficacy. However, the antibiotic treatment reduced the efficacy of AP from 89.5 to 46.8% in microbiota-intact and microbiota-depleted mice, respectively. These findings demonstrate that the efficacy of AP for EC depends, at least partly, on the commensal gut microbiota (47). In addition, Yamamura et al. studied whether the high load of *F. nucleatum* in the tumor of ESCC patients is related to the efficacy of neoadjuvant chemotherapy. All patients were pathologically evaluated with imaging data provided by CT scans, metabolic response rates determined by the maximum standardized uptake value from PET/CT imaging, and tumor regression grade analysis. The results showed that patients with high loads of *F. nucleatum* in tumors seemed to be more resistant to neoadjuvant chemotherapy treatment (85); therefore, the use of narrow-spectrum antibiotics to eradicate *F. nucleatum* might increase the effectiveness of neoadjuvant chemotherapy in ESCC patients.

Considering that the effect of microbiota on EC plays a role in the immune system, it is reasonable to presume that microflora may also affect the response of their hosts to immunotherapy. Immunotherapy is a valid method for treating many types of cancer and has become a complementary method after surgical treatment, radiotherapy, and chemotherapy. Immune checkpoint inhibitors can reactivate tumor-reactive T cells to induce antitumor immune responses (117). The normal gut microbiota are necessary for an effective immune response caused by programmed cell death 1 or programmed cell death ligand 1 (118–121) and cytotoxic T lymphocyte-associated antigen-4 (122). Previous studies have reported that *Akkermansia muciniphila* (120), *Eubacterium limosum* (123), and *Alistipes shahii* (55) were positively associated with immunotherapeutic effects. Importantly, a study has reported that the use of antibiotics can weaken the effect of immunotherapy by CpG oligonucleotides in mice with subcutaneous tumors such as EC (55).

### Harnessing Microbiota to Prevent Therapy-Related Complications

Interestingly, in addition to the prevention and treatment of EC itself, gut microbiota is also related to complications in the treatment process, which fully demonstrates the close relationship between gut microbiota and EC. For instance, Tanaka et al. found that the duration of the systemic inflammatory response syndrome after esophagectomy was significantly shorter in the synbiotic group than in the control group (51). In addition, Giamarellos-Bourboulis found that administering synbiotics can reduce endotoxin, white blood cell count, C-reactive protein level, and the incidence of septic complications (124, 125) after EC surgery. This may be because the modification of intestinal microflora with synbiotics can weaken the overgrowth of bacteria in the intestine and reduce the translocation of bacteria to distant organs (126, 127). Moreover, Okada et al. reported that *Bifidobacterium* can reduce the expression of proinflammatory cytokines, which may be related to the inhibition of Ikappa B-α phosphorylation. These results suggest that rebuilding beneficial gut microbiota may reduce excessive inflammatory responses through direct immunomodulatory effects (128).

In addition to preventing complications from surgery, modulating the microbiota can also reduce the incidence of complications caused by esophageal chemotherapy. For instance, Masaaki et al. found that synbiotics distinctly weakened the severity of lymphopenia after EC chemotherapy (12), because synbiotics sustained the gut microbiota and the relatively low pH, which improved the nutritional status of colonocytes. Most importantly, relevant studies have revealed that the gut microbiota has potential application either for enhancing the efficacy of treatment or for preventing complications. More research is needed to assess the potential of these corresponding microbiota for clinical applications in the context of EC.

## Current Challenges and Future Prospects

Extensive studies have identified the importance of EC microbiota, which interact closely with host esophageal epithelial cells and play an important role in the development of EC and in the elucidation of the mechanisms of carcinogenesis. Hence, there are unprecedented opportunities to find new ways of diagnosing and treating EC, and there is a scientific challenge in identifying different biomarkers. In the past, oral and esophageal microflora were mainly studied as predictive markers of EC (30, 48). However, in addition to the oral cavity and esophagus, convenient samples can also be obtained from feces; thus, finding predictive markers of EC in feces requires further research. It is hoped that in the near future, relevant microflora will be found, and large randomized controlled trials will be conducted to prove their efficacy. In addition, the success of fecal microflora transplantation in treating recurrent *Clostridium difficile* has stimulated considerable interest in manipulating gut microbiota, but there is currently little consensus on the best intervention approach (129). Therefore, efforts need to be made to find the best way to intervene and manipulate the gut microbiome. In addition to fecal microflora transplantation, several advantageous microbial species can be administered as probiotics, and the direct clinical benefits and associated microbial benefits in EC also remain to be determined. In addition, studies on the influence of intestinal microorganisms on the prognosis of EC mainly focus on the prediction of EC stage, and it is important to establish an intestinal flora model to predict the sensitivity of radiotherapy, chemotherapy, and immunotherapy for EC and the incidence of postoperative complications. The use of probiotics to reduce complications after esophagectomy and chemoradiotherapy requires additional supporting data from the trial, and efforts are needed to further understand the carcinogenic effects of gut microbes and the mechanisms that regulate tumor response to treatment. With developments in this rapidly evolving field, the microbiota will be an important part of cancer prevention and treatment.

## Conclusion

**Figure 4:**
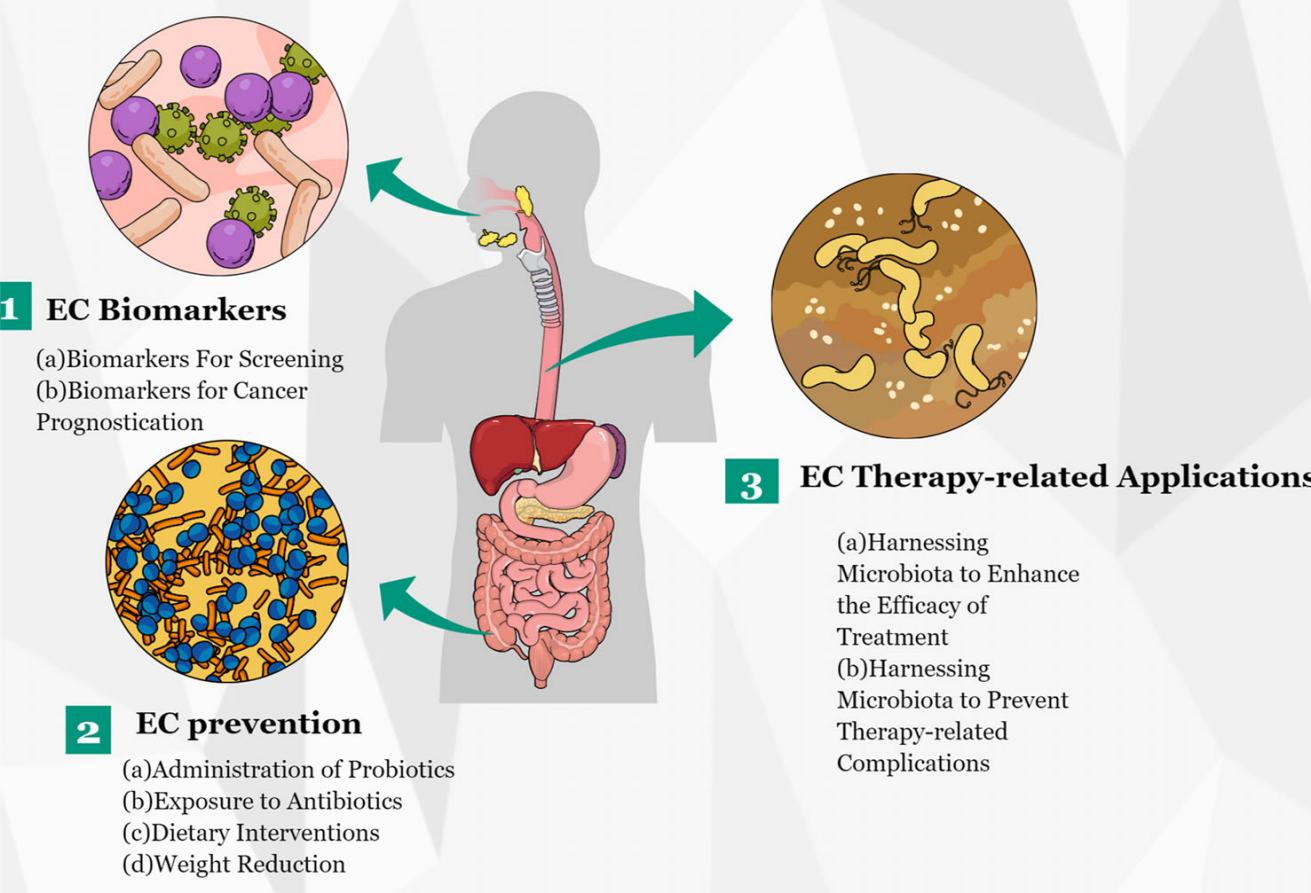
Potential clinical applications related to gut microbiota in EC. Several potential clinical applications for harnessing the gut microbiota in EC are depicted and include the development of screening, prognostic, and predictive biomarkers, and microbiota modulation for EC prevention and treatment.

In the last several years, a growing number of studies has revealed the vital role of microbiota in EC and have suggested that an imbalance in gut microbiota might lead to esophageal tumorigenesis. In addition, several research groups have conducted functional studies to verify the role of individual microbiota in carcinogenesis. In summary, these reported results offer an unprecedented opportunity for the translation of microbiota discoveries to clinical applications (**Figure 4**). With the progress of research in EC metagenomics and metabolomics, microbiota discoveries will potentially enrich treatment modalities for EC in the near future.

## Author Contributions

YY conceptualized the study, revised the manuscript, and supervised the study. JZ and SS conceptualized the study, drafted the manuscript, and made the figures. SL, XX, YSY, CM, LC, XZ and YZ collected the literature and revised the manuscript. All authors contributed to the article and approved the submitted version.

## Funding

This study was supported by the National Natural Science Foundation of China (Grant No. 81970481) and 1.3.5 project for disciplines of excellence, West China Hospital, Sichuan University (Grant Nos. 2020HXFH047 and 20HXJS005).

## Conflict of Interest

The authors declare that the research was conducted in the absence of any commercial or financial relationships that could be construed as a potential conflict of interest.

## Publisher’s Note

All claims expressed in this article are solely those of the authors and do not necessarily represent those of their affiliated organizations, or those of the publisher, the editors and the reviewers. Any product that may be evaluated in this article, or claim that may be made by its manufacturer, is not guaranteed or endorsed by the publisher.

## Abbreviations

BE, Barret’s esophagus; EAC, esophageal adenocarcinoma; EC, esophageal cancer; ESCC, esophageal squamous cell carcinoma; GERD, gastroesophageal flux disease; LPS, lipopolysaccharide; NOD, nucleotide-binding oligomeric domain; RE, reflux esophagitis; rRNA, ribosomal RNA; TLR, Toll-like receptors; ROS, reactive oxygen species.
